# Leveraging geo-referenced malaria information to increase domestic financial support for malaria elimination in Thailand

**DOI:** 10.1186/s12936-022-04227-1

**Published:** 2022-07-07

**Authors:** Prayuth Sudathip, Pratin Dharmarak, Sara Rossi, Nutthawoot Promda, Gretchen Newby, Erika Larson, Deyer Gopinath, Jerdsuda Kanjanasuwan, Praparat Promeiang, Cheewanan Lertpiriyasuwat, Darin Areechokchai, Preecha Prempree

**Affiliations:** 1 Division of Vector Borne Diseases, Department of Disease Control, Ministry of Public Health, Nonthaburi, Thailand; 2 Malaria Elimination Initiative, University of California San Francisco, San Francisco, CA USA; 3 World Health Organization, Nonthaburi, Thailand; 4 Department of Disease Control, Ministry of Public Health, Nonthaburi, Thailand

**Keywords:** Malaria elimination, Thailand, Financing, Domestic resource mobilization, Budget advocacy, Capacity building, Malaria information system, Multisectoral collaboration, Stratification, Vector control

## Abstract

Thailand’s National Malaria Elimination Strategy 2017–2026 seeks to increase domestic support and financing for malaria elimination. During 2018–2020, through a series of training sessions, public health officials in Thailand utilized foci-level malaria data to engage subdistrict-level government units known as Local Administrative Organizations (LAOs) with the aim of increasing their understanding of their local malaria situation, collaboration with public health networks, and advocacy for financial support of targeted interventions in villages within their jurisdictions. As a result of these efforts, total LAO funding support for malaria nearly doubled from the 2017 baseline to 2020. In 2021, a novel “LAO collaboration” feature was added to Thailand’s national malaria information system that enables tracking and visualization of LAO financial support of malaria in areas with transmission, by year, down to the subdistrict level. This case study describes Thailand’s experience implementing the LAO engagement strategy, quantifying and monitoring the financial support mobilized from LAOs, and results from a qualitative study in five high-performance provinces examining factors and approaches that foster successful local collaboration between LAOs, public health networks, and communities for malaria prevention and response. Results from the study showed that significant malaria endemicity or local outbreaks helped spur collaboration in multiple provinces. Increases in LAO support and involvement were attributable to four approaches employed by public health officials: (a) strengthening malaria literacy and response capacity of LAOs, (b) organizational leadership in response to outbreaks, (c) utilization of structural incentives, and (d) multisectoral involvement in malaria response. In two provinces, capacity building of LAOs in malaria vector control, following a precedent set by Thailand’s dengue programme, enabled LAO personnel to play both funding and implementation roles in local malaria response. Wider replication of the LAO engagement strategy across Thailand may sustain gains and yield efficiencies in the fight against malaria as the vector-borne disease workforce declines. Lessons from Thailand’s experience may be useful for malaria programmes in other geographies to support the goals and sustainability of elimination and prevention of re-establishment by improving financing through local collaboration between the health system and elected officials.

## Background

**Fig. 1:**
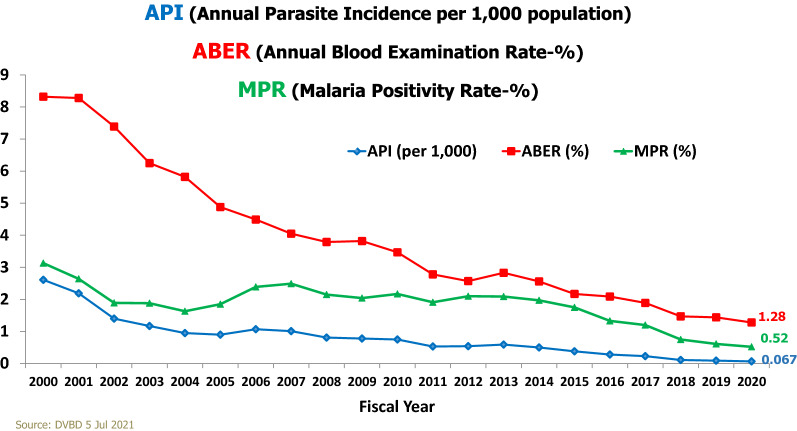
Trends in Thailand’s malaria incidence, 2000–2020

**Fig. 2:**
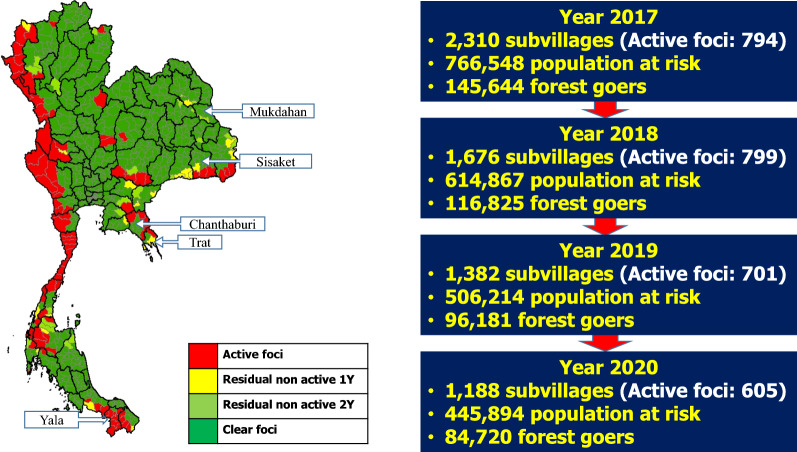
Active malaria transmission foci in Thailand, 2020

Since 2000, Thailand has made significant progress in reducing its malaria burden. The country has had an annual parasite incidence (API) of less than 0.1 per 1,000 population at risk since 2008 (Fig. 1) [1]. During Thailand’s fiscal year 2020, only 4,423 malaria cases were reported, of which 3,487 (79%) were indigenous. The number of active transmission foci has been in steady decline for the past several years, with 605 foci nationwide in 2020 (Fig. 2). There is heterogeneity of transmission between provinces and districts. Remaining pockets of transmission are mostly located along mountainous border areas where prompt access to diagnosis and treatment and timely follow-up with comprehensive measures remain challenging.

Thailand’s national malaria programme, the Division of Vector Borne Diseases (DVBD), which is part of the Department of Disease Control (DDC) within the Ministry of Public Health (MoPH), developed a 10-year National Malaria Elimination Strategy (NMES) 2017–2026 to guide the country’s malaria programming and support the long-term sustainability of malaria elimination and prevention of re-establishment. Thailand aims to stop transmission of *Plasmodium falciparum* malaria by 2023 and achieve zero local transmission of all malaria by 2024. To facilitate this goal, the DVBD utilizes a dynamic, web-based malaria information system called Malaria Online. In use since 2012, Malaria Online tracks geospatial, case-based entomological, epidemiological, and laboratory surveillance data in real time. The system generates automated, aggregated, and graphical reports, allowing the DVBD to update foci classification on an annual basis and as cases are reported during the year [2, 3]. Foci are assigned to one of four categories of transmission, with different prescribed interventions for each (Table 1). Foci classified as A1 and A2 are considered at risk of malaria, with active or residual transmission [4, 5].

**Table 1:** Foci classification in Thailand and key interventions

Foci classification	Current definition	Key interventions targeted at index households and at-risk neighbors
A1	Active foci: village or subvillage with indigenous cases in the current year	Passive case detection, radical cure of all cases, 1–3-7, iDES, active case detection twice a year, vector control by IRS or ITN, health education on malaria
A2	Residual non-active foci: village or subvillage without indigenous cases in the current year but with indigenous cases in the previous 3 years	Passive case detection, radical cure of all cases, 1–3-7, iDES, active case detection once a year, vector control by IRS or ITN, health education on malaria
B1	Cleared foci but receptive: village or subvillage without indigenous cases for 3 consecutive years but vectors are found or environment is suitable for vector breeding	Passive case detection, radical cure for all cases, 1–3-7 for index cases
B2	Cleared foci but not receptive: village or subvillage without indigenous cases for 3 consecutive years but vectors are not found or environment is not suitable for vector breeding	Passive case detection, radical cure for all cases, 1–3-7 for index cases

*iDES* integrated drug efficacy surveillance, *IRS* indoor residual spraying, *ITN* insecticide treated net

In addition to hyper-local information on (sub)village-level malaria foci, which enables interventions to be targeted to the index household and other at-risk neighbours, Malaria Online also contains information on subdistrict-level stratification, which corresponds with the jurisdiction of government units called Local Administrative Organizations (LAOs). Beyond activity planning and monitoring, public health officials utilize Malaria Online’s location-specific surveillance data as the basis for advocacy with LAOs to mobilize support for necessary prevention and response measures.

During 2018–2020, public health officials in Thailand strengthened local ownership and financial support for malaria elimination through a series of capacity-building trainings and engagement of local stakeholders at operational level. These efforts were aimed at LAOs as well as the public health network, comprised of the vertical vector-borne disease programme and staff in the general health services (GHS) system. Strengthening collaboration to mobilize and access resources through a multistakeholder approach required working across management streams within the MoPH, with other entities such as the National Health Security Office (NHSO), and across government ministries and levels of public administration. This case study provides contextual information and documents implementation of the LAO engagement strategy, quantification of financial support from LAOs and creation of a novel Malaria Online dashboard for domestic finance tracking, and findings from five high-performance provinces on strategies and approaches for effective engagement with LAOs.

### Malaria service delivery and funding

Under the MoPH, there are two service delivery systems for malaria: the vertical malaria programme and the GHS system. In the vertical programme, the DDC supervises regional Offices of Disease Prevention and Control (ODPCs), provincial Vector Borne Disease Centers (VBDCs), and district-level Vector Borne Disease Units (VBDUs) that have malaria clinics attached. These VBDUs/clinics are responsible for malaria-specific activities including prevention education, vector control, diagnosis and treatment, surveillance, and recording and reporting data in Malaria Online. However, the number of vector-borne disease programme staff is in continual decline, as MoPH policy is to not replace those who retire. In response to closure of VBDUs and clinics in some areas, the national malaria programme has provided training and malaria commodities to hospitals and public health offices under the GHS system at provincial, district, and subdistrict levels to capacitate them to deliver high-quality case management services, while VBDCs continue to conduct and support vector control services and active case finding as needed.

It is envisioned that the vertical malaria programme will be phased out eventually, maintaining only limited specialized tasks such as entomological surveillance and provision of specific technical support to the GHS and other relevant entities, including LAOs. Thailand’s intention to integrate malaria programming into the GHS underscores the need to define clear roles and responsibilities among all stakeholders, while maintaining and strengthening technical and programmatic capacity for analysis and decision-making at subnational level.

Like other countries in the Greater Mekong Sub-region (GMS), Thailand’s financial resources in the fight against malaria have come from a combination of national budgets and donor funding. The Global Fund to Fight AIDS, Tuberculosis and Malaria (Global Fund) has provided most of Thailand’s donor funding for malaria since 2004, directing resources to the DDC. Thailand also receives financial and technical assistance from the U.S. President’s Malaria Initiative (PMI), the World Health Organization (WHO), and other partners for malaria commodities and other programme needs. Due to the country’s relatively low malaria burden, upper-middle income status, and with elimination in sight, it is anticipated that Thailand will eventually transition from Global Fund support for its malaria response and may face significant declines in donor funding after the current regional grant ends in 2023. Although Thailand hopes to eliminate local malaria transmission within one year of that end date, adequate technical capabilities, surveillance systems, and commodities must be maintained to prevent re-establishment. Thus, domestic sources of funding must be identified and mobilized to support sustainability of Thailand’s malaria response.

The need for increased domestic financing is reflected in the NMES. One of the four key priorities laid out in the strategic plan is to “foster collaboration and partnership among stakeholders at national and international levels in order to enable malaria elimination,” which involves efforts to “motivate/advocate to partners to invest and share resources” [6]. Within this measure are two priority activities: (a) develop capacity of partners’ personnel in playing roles in malaria elimination, and (b) motivate institutions under the Department of LAO to play a role in malaria prevention and control.” The NMES indicator associated with these activities is the percentage of LAO offices in areas at risk of malaria transmission that implement (i.e., financially support) malaria elimination.

### Thailand’s local governance system, roles, and resources

**Fig. 3:**
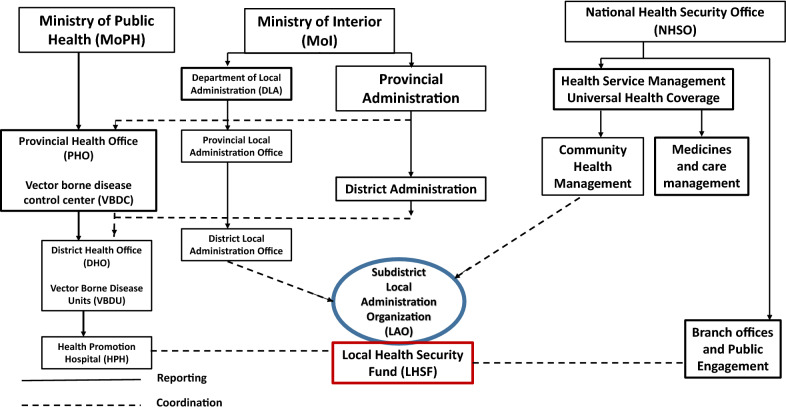
Health-related public administration structure in Thailand

The Thai public administration system comprises three administrative levels (Fig. 3). These include the central level with over twenty ministries and agencies in Bangkok, the regional level that includes provinces and districts, and local level which includes subdistricts, called *Tambon*, that are administered by LAOs. LAO leaders are a mix of locally elected officials who serve four-year terms and whose mandate is to make decisions on local plans (these include the chief executive, deputy chief executive, and *Tambon* council members), and permanent government officials in the Ministry of Interior (MoI) civil service who perform legal, financial, and managerial tasks for public and community services.

The responsibilities of LAOs include provision of public infrastructure; improving and promoting public health and welfare; local social order and security maintenance; economic development; management of natural resources and environment; and local cultural promotion [7]. An LAO’s financial resources are generated through local taxes and revenues, annual allocations from MoI, and area-based special purpose funds, including the Local Health Security Fund (LHSF), which is also sometimes referred to as the Community Health Fund, Local Health Fund, *Tambon* health fund, or *Tambon* Health Insurance Fund.

### LAO roles in health and LHSF administration

LAOs support some local health services and activities through three funding channels: their annual budget plans, special programmes such as the Royal Initiative for Public Health Fund, and the LHSF—which, as one of the largest of LAO budget sources for health promotion and disease prevention, is the cornerstone of the DVBD’s strategy for LAO engagement, collaboration, and resource mobilization. There are advantages, disadvantages, and limitations inherent in each type of health funding available to LAOs. Utilization of any of these budget sources is at the discretion of LAO officials.

The LHSF is a fund-pooling mechanism for community-based health promotion and disease prevention. It was introduced in 2007 as part of Thailand’s implementation of universal healthcare coverage and scaled up as more LAOs opted into the initiative. Among other health-related purposes, LHSFs are meant to “support disease prevention and control in case of outbreaks and disaster as necessary in a timely manner” [8]. They are resourced through a prevention and promotion area-based payment from the National Health Security Office (NHSO), the agency responsible for administering Thailand’s universal healthcare coverage scheme—45 Baht per capita per year, amounting to US $118 million total. This is matched by funds from the LAO, ranging from 30%-50% depending on the LAO’s size and capacity [9].

Each LHSF is managed by a committee that meets periodically to review and approve project proposals and includes representatives from the LAO council, village health volunteers (VHVs), local health officials, community leaders, and health workers. Eligible applicants for LHSF funding include local health agencies, service providers, and groups of volunteers with requisite experience and capabilities to utilize the funds to address local public health issues that have been discussed and prioritized through community meetings [10, 11].

During many decades of malaria programme implementation in Thailand, the tasks of programme design and resource mobilization have largely been the responsibility of government administration at central level. As such, promoting the use of LHSF funds for malaria elimination projects required strengthening knowledge and capacity of LAOs to facilitate improved and scaled collaboration, and agreement among agencies with health mandates on their respective roles and responsibilities in local malaria foci response.

### Implementation of the LAO engagement strategy

DVBD engagement of LAOs for malaria support had three objectives: (a) to increase the amount of malaria activity funding contributed by LAOs, (b) to increase the percentage of LAOs contributing to malaria activities in areas with transmission, and (c) to make LAO support data visible to the malaria service delivery network and the public through a Malaria Online dashboard.

**Fig. 4:**
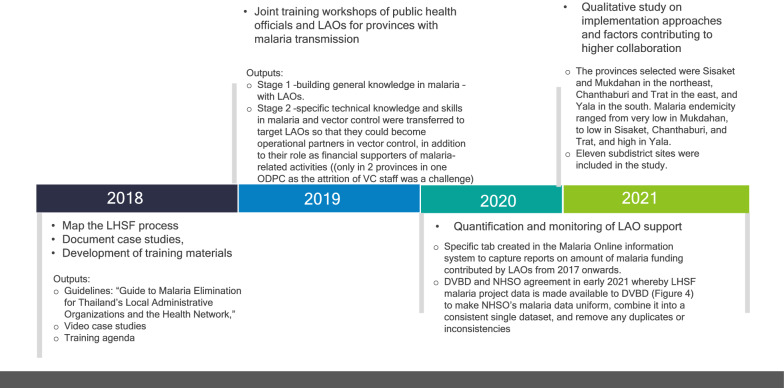
Timeline of key activities in LAO engagement, 2018–2021

Key activities involved fieldwork to map the LHSF process and document case studies, development and dissemination of training materials including an agenda for training-of-trainers (ToT), quantification and monitoring of financial support received, establishment of data visualization systems for ongoing tracking, and research in five provinces with high levels of LAO support to understand key facilitating factors for collaboration (Table 2 and Fig. 4) [12]. The latter three activities are described in detail below.

**Table 2:** Objectives and activities comprising DVBD’s LAO engagement strategy

Objectives	(a) Increase amount of malaria activity funding contributed by LAOs	(b) Increase the percentage of LAOs contributing to malaria activities in areas with transmission	(c) Make LAO support data visible to the malaria service delivery network and the public
Activities	•Map the LHSF process and document case studies (written and video) •Develop and disseminate training resources via ToT •Research to understand key facilitating factors for collaboration		•Quantify and monitor financial support received •Establish data visualization systems for ongoing tracking

### Quantification and monitoring of LAO support

The DVBD set up a database in its Malaria Online system, into which PHO programme assistants in provinces with transmission can report the amount of malaria funding contributed by LAOs from 2017 onwards. To ensure comprehensiveness of LAO contribution data, an agreement was negotiated between DVBD and NHSO in early 2021, whereby LHSF malaria project data is made available to DVBD in Microsoft Excel format for upload to the DVBD server.

The amounts reported through the end of fiscal year 2020 from NHSO and PHOs showed a significant increase in the level of support, despite decreasing malaria burden: there was an 87% increase in LAO contributions from 2017 to 2020 (Table 3), from 3,714,999 Baht (US $110,972) in 2017 to 6,945,273 Baht (US $207,464) in 2020. The percentage of A1/A2 subdistricts with financial support from LAOs nearly quadrupled, from 10% in 2017 to 38% in 2020. The results indicated that some provinces had higher contributions than others (Table 4).

**Table 3:** Amount of funding and % of A1/A2 subdistricts supported by LAOs during fiscal year 2017–2020

Year	**2017**	**2018**	**2019**	**2020**	**Total**
Amount in Thai Baht	3,714,999	4,489,771	6,130,855	6,945,273	21,280,898
% of LAOs with A1/A2 subdistricts providing support	10%	18%	31%	38%	
Number of provinces with malaria transmission	46	41	42	39

**Table 4:** Top 10 provinces with high % of LAO contributions to malaria, among subdistricts categorized as A1/A2

Rank	Province	2017	2018	2019	2020
1	Yala	80%	75%	80%	68%
2	Prachin Buri	0%	100%	100%	67%
3	Prachuap Khiri Khan	43%	53%	60%	67%
4	Mukdahan	13%	14%	92%	90%
5	Chanthaburi	14%	67%	63%	63%
6	Rayong	0%	100%	100%	0%
7	Chiangrai	18%	57%	75%	50%
8	Lampang	0%	0%	75%	100%
9	Trat	40%	18%	50%	67%
10	Krabi	0%	0%	67%	100%

### Establishment of data visualization for tracking

**Fig. 5:**
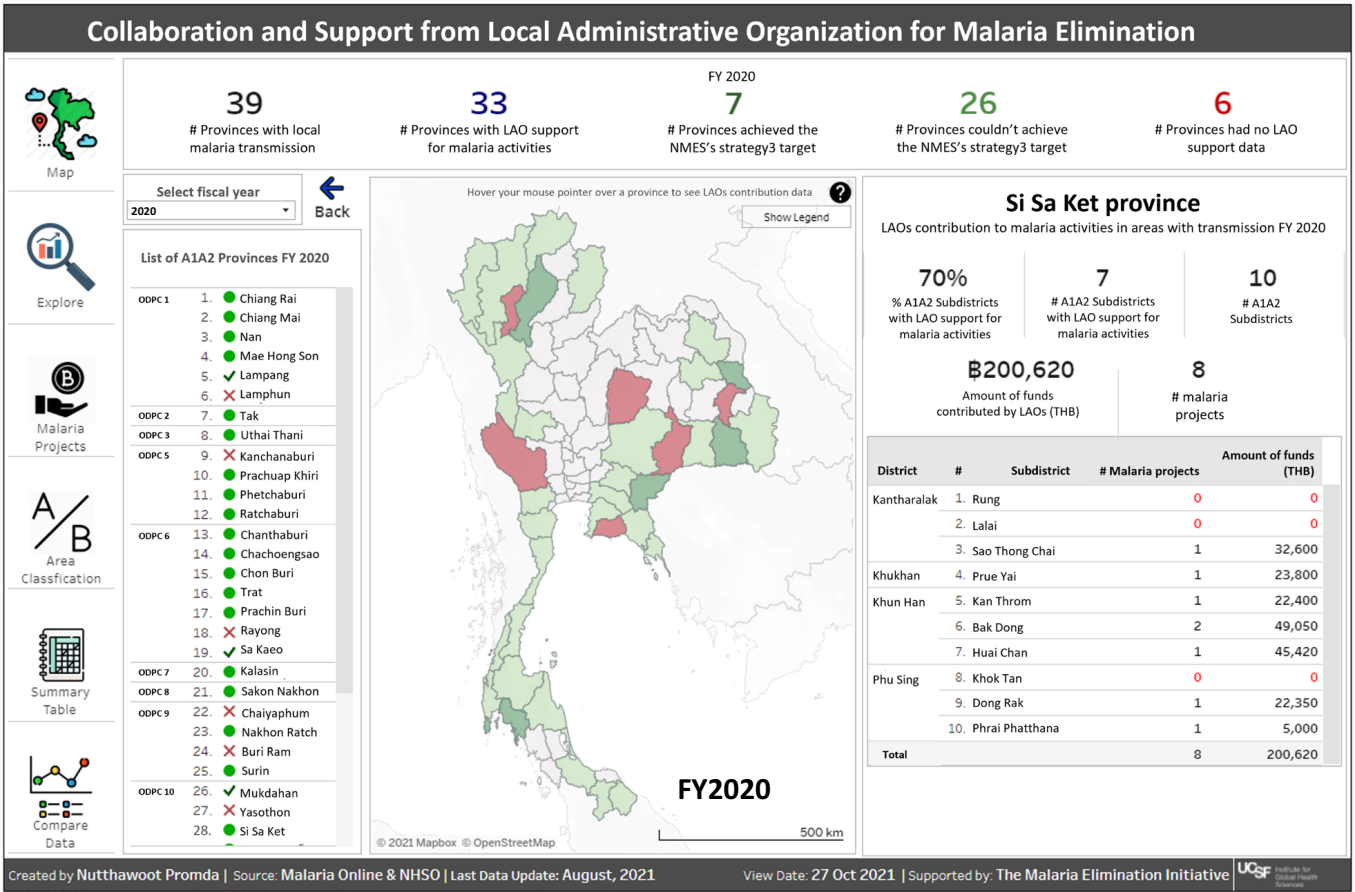
“LAO Collaboration” Tableau dashboard in Malaria Online

A data visualization dashboard was designed in Tableau that allows users to see LAO funding (amounts and project names) for malaria by province, district, and subdistrict, paired with transmission and stratification data by year. The dashboard was launched as a new feature of Malaria Online, accessible to all users, in June 2021. In the dashboard, a color-coded view of provinces with high and low collaboration is shown, with green indicating higher % of collaboration and red indicating lower % of collaboration (Fig. 5). When the user hovers the mouse over a province, information on that province is shown, including a list of its A1/A2 subdistricts and a list of LHSF projects and funding amounts. By clicking on a province, detailed information about the province’s A1/A2 subdistricts—e.g., amount of support and source of funding by year—is shown.

### Qualitative study on implementation approaches and factors contributing to higher collaboration

#### Objective

To inform ongoing LAO advocacy and training efforts, DVBD sought to understand key contextual factors and approaches that have contributed to high levels of LAO support in certain provinces through qualitative research undertaken in five provinces in 2021.

#### Methods

The study purposively selected five high-performing provinces that have demonstrated significant LAO contributions to malaria elimination activities (Table 5) and have a wide range of malaria endemicity. Thailand’s Covid-19 situation created travel difficulties and, therefore, limited the number and choices of provinces. The provinces selected were Sisaket and Mukdahan under ODPC 10 in the northeast, Chanthaburi and Trat under ODPC 6 in the east, and Yala under ODPC 12 in the south. Malaria endemicity ranged from very low in Mukdahan, to low in Sisaket, Chanthaburi, and Trat, and high in Yala. Eleven subdistrict sites were included in the study.

**Table 5:** Percent of LAOs in A1/A2 subdistricts with malaria financial contributions and amount in Thai Baht, FY 2017–2020, for provinces included in the qualitative study

ODPC	Province		2017	2018	2019	2020	Total amount (Thai Baht)
6	Chanthaburi	% A1/A2 subdistricts with contributions	14%	67%	63%	63%	
		Amount	29,000	291,150	272,500	244,746	837,796
	Trat	% A1/A2 subdistricts with contributions	40%	18%	50%	66%	
		Amount	211,200	66,000	154,345	181,022	612,567
10	Sisaket	% A1/A2 subdistricts with contributions	N/A	30%	27%	70%	
		Amount		39,100	100,060	200,620	339,780
	Mukdahan	% A1/A2 subdistricts with contributions	13%	14%	92%	90%	
		Amount	76,000	64,960	363,100	292,700	796,760
12	Yala	% A1/A2 subdistricts with contributions	80%	75%	80%	72%	
		Amount	1,720,632	1,624,525	1,442,353	1,906,104	6,693,614

Data collection was conducted in the five provinces from January to April 2021, during which 40 key informants were interviewed on-site at ODPCs, VBDCs, VBDUs, PHOs, health promotion hospitals (HPHs), LAOs, and Regional Health Security Offices using a semi-structured qualitative interview guide (Table 6). Three additional key informants were interviewed using the same tool through video calls. Transcribed interview data was analysed by province to understand the LAO engagement implementation process as individual case studies. Subsequently, a comparative cross-case analysis was conducted, and data was organized into four themes.

**Table 6:** List of key informants involved in LAO engagement study

Agency/affiliation	Abbreviation	Number	Codes
Office of Disease Prevention and Control	ODPC	3	A, B, C
Vector Borne Disease Center	VBDC	4	1–4
Vector Borne Disease Unit	VBDU	4	1–4
Provincial Health Office	PHO	5	1–5
District Health Office	DHO	3	1–3
Health promotion hospital	HPH	6	1–6
Village Health Volunteer	VHV	3	1–3
Civil society organization	CSO	1	1
Military	Mil	1	1
Local Administrative Organization	LAO	11	1–11

Key informants (KIs) from both the public health side and the LAOs were asked, “What do you think are the factors contributing to the increase in collaboration between health agencies and LAO for malaria elimination in your province?” Responses were categorized into four major themes: (a) strengthening malaria literacy and response capacity of LAOs; (b) organizational leadership in response to outbreaks; (c) utilization of structural incentives; and (d) multisectoral involvement in malaria response.

#### Results

##### Increased malaria awareness and capacity and use of stratification for focused investment enable LAOs to provide financial and political support in all provinces, and take on new service delivery roles in two provinces

KIs in all five provinces reported building capacity of LAO officials and personnel as the most important mechanism to facilitate LAO-public health collaboration on malaria. The LAO capacity building approach consists of two stages: efforts by public health officials to provide LAOs with information about the local malaria situation, required interventions according to stratification, and implementation arrangements for LAO funding (Stage 1); and the process of transferring technical knowledge and skills to LAOs or affiliated local malaria teams so that they can perform vector control tasks as an operational partner of local VBDUs (Stage 2). Stage 1 capacity building was reported in all provinces and was executed through the joint training workshops. Stage 2 capacity building activities are occurring in two provinces under ODPC #A. Respondents reported that increased awareness and understanding of malaria among LAO staff and LHSF Committees motivates them to support malaria projects proposed for LHSF funding or other LAO budgetary support. Having shared access to and understanding of stratification of malaria risk at foci level helps vector-borne disease staff present a strong case for targeted investment to LHSF Committees and later link positive outcomes of fewer or no malaria cases to the local response funding.

Example accounts of Stage 1 LAO capacity building:

“I went to a workshop in Bangkok with PHO in 2018 and we learned that the LHSF fund can be used to support malaria activities. Since then, we have been working with both HPH and VBDU to get rid of malaria in our subdistrict.” (LAO #4).

“The HPH informed us about the malaria situation and about local groups of people who are at-risk of getting malaria. We need to review the proposed activities in detail, the intended results, and see that the costs are reasonable.” (LAO #6).

“The VBDU staff always come to meet us and give us information about malaria and what needs to be done. We have good relationships with the VBDU. The budget provided will benefit local villagers to have better health.” (LAO #2).

“We informed the LAO that there are still specific clusters of a few households with active transmission (A1 stratification) that require interventions. As the area is not large, required activities and the amount of funding is not very high; these factors helped the LAO to make the decision to implement the plan rather easily.” (VBDU #3).

The Stage 2 capacity building approach, which aims to have LAOs become co-implementers or operational partners in malaria elimination and prevention of re-establishment, is being employed by four subdistricts within two provinces under ODPC #A. In these subdistricts, VBDU staff work with their LAO counterparts to carry out necessary malaria vector control interventions according to local transmission risk, as designated by stratification, for a period of three years. In particular, LAOs have been trained in some subdistricts to carry out IRS spraying and treatment of bednets, similar to the vector control role Thailand’s LAOs have taken on in the fight against dengue fever.

In the past, many vector-borne disease agencies under the jurisdiction of ODPC #A received funding support from LAOs for vector control-related tasks. Since 2017, the approach has shifted following guidance of the provincial VBDC and ODPC. District-level VBDU staff now work with LAO personnel to prepare project documents for review by LHSF Committees and LAO executives. After approval, LAOs serve two roles: they manage project implementation by volunteers, and pay the costs of chemicals and labor for IRS spray campaigns. In this arrangement, VBDU staff continue to serve as co-implementers of activities and technical trainers to the LAO. They also monitor the quality of work and report the activities into Malaria Online.

“In the past, each agency worked on their own. After the joint training, we understood more about malaria. We then tuned in to each other: the LAO has increased support for malaria activities and works together with other health agencies as a team. LAOs like us do not have technical knowledge and skills in malaria, so we have to work with the VBDU who provides us with information on the number of households that need IRS, what chemicals to use, and how many workers we need. We have a team of volunteers that have been trained to conduct the spraying.” (LAO #1).

Through the consistent, proactive efforts of public health staff to build shared malaria and LHSF understanding among LAO personnel, community members, and other stakeholders, collaborative relationships and local ownership have led to strong support.

##### Public health leaders have taken opportunities during periods of high malaria transmission to motivate staff, and expand and strengthen collaborative partnerships for malaria control and elimination

Discussions and interviews with KIs revealed strategic actions taken by public health personnel from ODPCs, PHOs, and VBDCs, who seized the moment when malaria cases spiked or local epidemics broke out to mobilize efforts and tackle malaria in varying ways.

A notable effort was that of ODPC #A, which recognized a decline in the region’s malaria staffing and decided to develop vector control knowledge and skills in LAO staff, described above. During the years 2016–2017, there were a few malaria outbreaks in the four provinces under ODPC #A. In response to the surges, senior staff of the ODPC engaged VBDCs to mobilize needed support from LAOs and GHS agencies.

“We invited all 5 VBDCs to review the malaria situation and set up a plan for subdistrict malaria teams with specific designated roles for each partner, including indicators to monitor progress. More importantly, we wanted the roles of LAO in malaria elimination to be similar to dengue fever, for which vector control tasks have been implemented by LAOs. As our staff were decreasing, if we didn’t start expanding partnerships and adjusting our roles, we would lose the opportunity.” (ODPC #A).

ODPC #A also set up a monitoring and evaluation system for VBDC/VBDU to report monthly on collaboration with LAOs and other GHS agencies, in terms of financial support or their staff joining in malaria response activities such as 1–3-7 tasks. When the DVBD launched its LAO engagement strategy in 2018, it reinforced at a national level the policy that ODPC #A had already been implementing; however, it did not go as far as ODPC #A in defining a role for LAOs in vector control.

PHO #3 executed its disease control mandate very impressively in response to malaria outbreaks. In 2017, abnormally high caseloads prompted the Provincial Communicable Disease Committee, with the PHO as secretariat, to convene a meeting of the Provincial Emergency Operation Center to raise public health attention and mobilize human and financial resources. Subsequently, the Provincial Governor appointed malaria response committees at provincial, district, and subdistrict levels. Annual joint meetings of the committees are convened by the PHO to update local stakeholders on the malaria situation and mobilize collaboration between public health officials, LAOs, forestry officials, and locally-stationed military personnel.

“It was very important to hold the joint meeting every year to inform local leaders of the malaria situation, analyse the problem, and devise a joint workplan. We wanted to increase understanding and the roles of LAO. Malaria is a problem of particular geography, and it is best that local people understand this, be the owner of the problem, and work out the solution. We have built a good network that regularly shares information when there are malaria cases and conducts the response together.” (PHO #3).

Because there is only one malaria clinic attached to a VBDU in this province, the PHO #3 took initiative to arrange training for hospital staff at district and subdistrict levels on malaria case management. This skill advancement among GHS staff has facilitated prompt access to high-quality malaria services for rural populations, and these efforts were praised by all stakeholders and local health officials. The PHO held joint trainings at provincial level that had high fidelity to the DVBD-led ToT trainings in Bangkok. Junior-level DHO and HPH staff felt that the support they received from PHO staff helped strengthen and expand their advocacy with LAOs at local level.

Public health officials, who are technical experts and career civil servants, have demonstrated sophisticated ability to engage LAOs as strategic political partners, and have figured out how to align incentives for mutually beneficial cooperation. A senior public health official working for the vector-borne disease programme in a province with very high malaria burden sees LAOs as strategic political partners to support the cause of malaria elimination.

“With increased understanding of malaria, LAOs can raise the profile of malaria and drive the elimination agenda in the community. However, we need to know how to work with LAOs. Being partners means we have to work together, alongside each other, and reciprocate each other’s needs. Around the year 2019, we had to target IRS spraying in… three connecting subdistricts. We did not have enough workers to do the task, so we worked with the three LAOs. We provided the chemicals and spraying equipment and requested the LAOs to provide 60 sprayers. We also held village-wide active case detection many times. LAOs made an announcement for villagers to come and provided the venues and lunches for all the malaria workers. When we asked an LAO to distribute LLINs to their constituents—they did that very efficiently and systematically.” (VBDC #4).

Seeing that a robust malaria response will improve the well-being of their local constituents, LAOs found ways to utilize their own resources, in addition to LHSF, to support necessary activities.

##### Some structural and administrative tools have been utilized to incentivize support from LAOs, but the results are not as sustainable as other mechanisms

In areas with very low malaria endemicity, gaining an LAO’s attention and investment in malaria can be challenging for public health officials because it can be considered an ‘out of sight, out of mind’ problem. In one province, structural mechanisms were employed to secure political and financial support from LAOs.

PHO #4 consulted with all its constituent districts that had subdistricts stratified as A1 or A2 to develop a strategy to garner LAO support through the District Health Board (DHB). The Board, with the District Health Officer as secretary, comprises representatives from the district government, leaders from other sectors such as education and business, and the District Local Administration (DLA) Officer. By adding malaria support as a DHB annual objective, the DLA Officer would transmit the objective down to the Subdistrict Health Board, which includes LAO representatives among its membership.

“At provincial level, we raised the importance of malaria elimination by adding malaria to the annual [Provincial Communicable Disease Committee] workplan for 2019–2020, and we added malaria to existing vector-borne workplans for dengue control. The contribution from LAOs in A1/A2 subdistricts was included as a key performance indicator for DHOs and other GHS agencies in all pertinent districts. We also requested support from the Provincial Local Administration Officer who relayed the message to the District Local Administration Officer.” (PHO #4).

When the PHO designated malaria support as a key performance indicator (KPI), it meant that the indicator had to be met by DHOs and subdistricts that year. Because the province had very low malaria burden, other competing health priorities, and limited resources, local public health staff from the DHO had to make the case through strong advocacy messages that malaria is an important disease requiring resources from LAOs to support prevention and active case detection activities proposed by HPH staff.

“When we requested support for malaria, we were asked in response if there is still malaria [in our community]. Other partners in the [DHB] did not see that it was a big problem. What I said to them was that, because the number of malaria cases is low compared to other diseases, this is the opportunity to eliminate the disease from our district, from our province. We are getting very close now, that’s why we need to work together on this and prevent it from coming back.” (DHO #3).

LAOs in this district were convinced of the benefits that would come from providing the requested malaria funding, including synergies with other vector-borne disease prevention efforts:

“The last malaria case in our district was in 2018. After that there have been no cases. However, we supported the project that was proposed by the HPH. We think people who are at-risk of malaria will have more knowledge about preventing malaria and reduce their risk. As malaria and dengue are vector-borne diseases, we supported dengue and malaria health education together in one project.” (LAO #8).

As a result of the strong incentive set by this PHO and efforts from the DHOs, during 2019 to 2020 the province achieved a very high percentage of LAOs in “A” subdistricts contributing funding for malaria response (90–100%). However, public health issue areas chosen as KPIs rotate regularly, and provincial attention and investment tend to follow. Lack of sustainability is a risk of using a time-bound, incentive-based approach.

##### Multisectoral partners have utilized village-level malaria data to heighten community awareness of malaria and catalyze actions from community leaders and members including support from LAOs

One of the five study provinces has a very high malaria burden, due to its favorable ecological conditions for malaria vectors and human behavioural factors including late treatment seeking and lack of treatment compliance. The province has also historically been affected by conflict and civil unrest, resulting in a large local military presence. In this province, army medical units provide health services to both military and civilian populations. Therefore, the province’s health authorities developed a multisectoral malaria response strategy wherein public health agencies, military personnel stationed in the area, and civil society organizations (CSOs) work together to raise community awareness and adopt necessary actions.

“In our role as medical personnel of the military, we have discussed the malaria situation with community leaders in villages with the highest malaria cases, emphasizing that it is a major concern and that we have to work together to change the situation. [Our malaria communication efforts target] LAO representatives, subdistrict chiefs, and religious leaders. This helped alert them to the severity of malaria in their areas.” (Mil #1).

“At the community forum held jointly by public health staff, the military, and CSOs, information about the local malaria situation came in from many sources. Everyone in the community knows that malaria is endemic here, including the LAO; as such, LAOs as local government authorities needed to take action to address the problem. No one could stand still and do nothing. We always emphasize that malaria can only be eliminated by the efforts and actions of all community members.” (CSO #1).

Local public health agencies and CSOs have mobilized a large network of VHVs and built their operational capacity to perform many tasks such as staffing malaria posts, communicating malaria risk, collecting blood films, and observing treatment. Strategic use of village-specific malaria data by all malaria stakeholders has heightened community awareness of the local malaria situation and resulted in active participation in malaria elimination efforts by community leaders as well as support from LAOs.

“Since early 2018, the objective of the ODPC has been to reduce malaria burden. It was very important to return malaria information back to the affected communities rather than keep epidemiological data accessible only to public health staff.” (ODPC #C).

To make malaria data available to partners and community members, the Disease Control Official of ODPC #C extracts provincial malaria surveillance data from Malaria Online and transforms it into a weekly malaria status update with simplified statistics listing Thailand’s top ten provinces in terms of number of malaria cases. The data is displayed in a bar graph format, showing the number of cases during the past 8 weeks by district, subdistrict, and village. VBDC #4 regularly uses this data for advocacy and decision-making with local health agencies and community leaders and shares key updates with the province’s “Malaria Network” chat group on the LINE mobile application that was set up to share timely information. The chat group has over 200 members comprising vector-borne disease, DHO, and HPH staff; community leaders; CSOs; VHVs; malaria post workers; military; and LAO personnel. News and pictures of community-based malaria activities carried out by malaria network partners (VHVs, VBDCs, and CSOs), such as reactive case detection, blood film collection, and treatment observation, are shared and discussed through this chat group on a daily basis.

In this province, LAO-funded malaria education vector control activities such as IRS have been increasingly carried out by groups of VHVs who organized themselves into subdistrict “Disease Beater Teams” that receive technical support from the district’s VBDU and local HPH. One representative of such a group reported:

“We always receive malaria situation updates from [the Public Health Technical Officer] from VBDC #4 through the chat group. This information is important for us to have and share with people in each village so that we know which location has malaria cases, as people always move between places in our province, and we need to be careful. It is important to know where we rank in the country—which district, subdistrict, and villages in our province still have malaria. [The Public Health Technical Officer] of VBDC #4 always gives us encouragement in our work as we make progress and less and less villages have malaria. Our team comprises not only volunteers, but also the village chief and the deputy. Before we do the IRS spraying as required for A1 and A2 villages, they help advise villagers to move their highly valued pet birds away for their safety and to bring them back afterwards. The LHSF Committee and the LAO executives do care about health problems that affect the population. When we presented our new project proposal to the LHSF Committee, we explained what we have done in the past and what were the results. In our subdistrict, we have fewer and fewer malaria cases in recently years, but we need to continue working hard, and the LAO is supportive of us.” (VHV #1).

“The local Disease Beater teams, most of whom are VHVs, have been working very well with support from community leaders and the LAOs. Seeing that malaria cases in their communities have decreased from interventions jointly implemented with VBDC, now the community-based teams take the needed actions without having to wait for us, and this should lead to sustainability. If we were asked what has made malaria in this province come down, the answer is—it is because all sectors have worked together.” (VBDC #4).

Collaborative efforts from all sectors in the province have had remarkable results in terms of driving down malaria incidence. At the time of the study in early 2021, the API of the province was at 0.3:1,000, compared to 1.9:1,000 in 2018—a decrease of 84% in three years.

## Discussion

### Increased malaria awareness and capacity and use of stratification for focused investment enable LAOs to provide financial and political support in all provinces and take on new service delivery roles in two provinces

Collaboration between public health agencies and LAOs predated the launch of Thailand’s 2017–2026 NMES and the DVBD-led LAO engagement strategy in several malaria-endemic provinces. ODPCs and provinces included in the study have responded to the occurrence of local outbreaks and shrinking malaria workforces by strengthening partnerships that would expand the human and financial resources available for malaria response. The official launch of the NMES with stratification for targeted malaria foci interventions and investment in 2016, the LAO engagement strategy in 2018, and the investment case for malaria elimination in Thailand in 2019 have led to a remarkable increase in engagement efforts across a broader array of provinces and significantly greater financial resources allocated by LAOs to malaria (Table 3) [13]. Foci-specific malaria information, when accessible and understood by affected communities and responsible authorities, increases sense of responsibility, ownership over the identification of problems and generation of solutions, and motivation to act.

Multisectoral involvement in malaria response in a high-burden setting reinforced and extended malaria elimination messages and information, which stimulated community-wide responses, including by the LAO. When malaria is more visible, it is easier to work with LAOs to prioritize malaria response and expand partnerships. When malaria is less of a concern, as in the lowest-burden provinces, structural mechanisms or incentives like KPIs have proven effective in boosting financial support from LAOs in the short-term but may not be as sustainable as other approaches. As Thailand is moving toward malaria elimination, besides working to increase domestic support, capacity building at sub-national levels has taken place among GHS personnel at district and subdistrict levels while malaria elimination courses for PHO level are underway, aiming to strengthen the capacity of local public health agencies in malaria elimination and prevention of re-establishment. The strategy is in close alignment with WHO’s newly updated Global Technical Strategy which emphasizes enhancing local capacity for malaria elimination and sustainability [14].

### Public health leaders have taken opportunities during periods of high malaria transmission to motivate staff and expand and strengthen collaborative partnerships for malaria control and elimination

Capacity building approaches varied across provinces. In all settings, public health officials engaged in Stage 1—or building general knowledge in malaria—with LAOs. Distinctively, the Stage 2 capacity building was implemented in the provinces under ODPC #A where specific technical knowledge and skills in malaria and vector control were transferred to target LAOs so that they could become operational partners in vector control, in addition to their role as financial supporters of malaria-related activities. This was due to the leadership and initiative of the supervising ODPC #A, who anticipated the province’s human resource needs as its vector-borne disease workforce progressively shrinks with retirements.

### Some structural and administrative tools have been utilized to incentivize support from LAOs, but the results are not as sustainable as other mechanisms

Though the population health mandate of LAOs is enshrined in national law, accessing LHSF funds for implementation of health promotion and disease prevention activities has proven difficult for many local stakeholders. The LHSF mechanism has often been underutilized due to complex budgetary rules, strict audit procedures, limited knowledge of health issues among community members to prepare project proposals, and inability of smaller LAOs to recruit suitable staff to manage the fund. LAOs may be reluctant to support new activities such as malaria response if they have not done so in the past or if they have less experience with the legal aspects and procedures to defend their decisions. This presents an opportunity for the national malaria programme (DVBD and DDC) to issue formal technical guidelines for new or expanded implementation roles of LAOs in vector control and the required capacity building involved, as well as allocating necessary resources for such undertakings. However, introducing new initiatives and plans will require strong support from policy level of the DDC and MoPH to ensure their success [15]. Moreover, as the spread of COVID-19 has resulted in movement restrictions for vector-borne disease staff, subdistrict capability to manage local malaria foci through vector control is even more critical. The COVID-19 pandemic has also forced most LAOs in Thailand to divert resources to COVID-19 emergency response. As long as the pandemic persists, the DDC and subnational malaria programme implementers will have to be focused and strategic about what to request from LAOs and how to maximize value for money.

The ability of each ODPC to build capacity of LAOs varies, as senior vector-borne staff in some ODPCs are retiring with few younger staff left. Nonetheless, relatively strong and capable cadres of vector-borne disease staff remain at both VBDC and VBDU levels. Technical training in situation analysis, basic entomology, and malaria vector control to LAOs should be possible once a clear policy directive on the roles of LAOs in malaria elimination is issued, with accompanying training packages and financial resources. As malaria burden decreases, it may be more practical, cost-effective, and sustainable to adopt a combined dengue and malaria approach for vector control, and to have it included in the Subdistrict Development Plan that will be the basis for actual budget allocation in the Annual Budget Plan of the LAOs [16]. To achieve this goal, the DDC will also need to have a formal agreement or MOU with the MoI DLA.

### Multisectoral partners have utilized village-level malaria data to heighten community awareness of malaria and catalyze actions from community leaders and members including support from LAOs

A relevant precedent is that LAOs across Thailand have taken on the tasks of vector control for dengue in collaboration with public health agencies since the year 2002. To support implementation, a Dengue Prevention and Control Manual for LAO developed by DVBD was disseminated in 2008 and multi-year cooperative agreements and MOUs between the DDC/MoPH and the DLA within the MoI have been signed periodically with specification of chemicals to be used, coordination between HPH and LAO, timing of related activities, reporting requirements, and LAO funding sources available to cover expenses. The MOUs are forwarded from the MoI DLA to LAOs in all provinces. To support malaria vector control sustainability at the subdistrict level, Thailand’s DDC could adopt a similar approach to dengue by setting clear policy guidance and building capacity of LAO for vector control in both diseases, or through integrated vector management where relevant.

## Conclusions

Stratification data from Malaria Online and an intentional capacity-strengthening effort led by the DVBD equipped public health agencies, LAO personnel, community actors, and other stakeholders with foci-specific malaria data, skills, and relationships to engage LAOs during 2018–2020 for sustainable collaboration and domestic resource mobilization for malaria elimination. Though accessing LAO resources for community-led malaria interventions was a core objective of the strategy, one of the key lessons learned from two innovative provinces is that the LAOs can be more than funders of malaria response—they can also act as an implementing partner in malaria elimination once adequate capacity is built. LAO involvement is also a good indicator of local coordination for disease prevention and control among pertinent stakeholders, even beyond malaria.

As Thailand prioritizes local ownership and domestic financing to achieve malaria elimination by 2024, the DVBD should continue supporting provinces, districts, and subdistricts to shift long-held roles of vertical vector-borne disease staff and empower subnational partners—including LAOs—to take on new roles in malaria elimination and prevention of resurgence. The LAO/LHSF example is unique to Thailand’s local governance, health system, and funding structures but serves as a good model and source of learnings for other malaria-eliminating countries—especially Thailand’s neighbors in the GMS. The need for local ownership and resource mobilization for malaria at the lowest administrative level is critical to foster sustainability while accelerating progress toward zero malaria.

## Data Availability

The visualizations supporting the conclusions of this article are available in the Malaria Online repository, http://malaria.ddc.moph.go.th/. The qualitative data from key informant interviews is not publicly available because participants did not consent to have their full transcripts made publicly available. However, excerpts of the transcripts relevant to the study are available upon reasonable request to the corresponding author.

## Abbreviations

API: Annual parasite incidence
CSO: Civil society organization
DDC: Department of Disease Control
DHB: District Health Board
DHO: District Health Office
DLA: District Local Administration
DVBD: Division of Vector Borne Diseases
GHS: General Health Services
Global Fund: The Global Fund to Fight AIDS, Tuberculosis and Malaria
GMS: Greater Mekong Sub-region
HPH: Health Promotion Hospital
iDES: Integrated drug efficacy surveillance
IRS: Indoor residual spraying
ITN: Insecticide-treated net
KI: Key informant
KPI: Key performance indicator
LAO: Local Administrative Organization
LHSF: Local Health Security Fund
Mil: Military
MoI: Ministry of Interior
MoPH: Ministry of Public Health
MOU: Memorandum of understanding
NHSO: National Health Security Office
NMES: National Malaria Elimination Strategy
ODPC: Office of Disease Prevention and Control
PHO: Provincial Health Office
PMI: U.S. President’s Malaria Initiative
ToT: Training-of-trainers
VBDC: Vector Borne Disease Center
VBDU: Vector Borne Disease Unit
VHV: Village Health Volunteer
WHO: World Health Organization
